# Breastfeeding duration and the risk of systemic juvenile idiopathic arthritis: a cross-sectional study

**DOI:** 10.3389/fnut.2025.1643926

**Published:** 2025-08-29

**Authors:** Qiang Luo, Hui Zhang, Xinlin Wu, Xiaoqiong Wei, Xuemei Tang

**Affiliations:** Department of Rheumatology and Immunology, Chongqing Key Laboratory of Child Rare Diseases in Infection and Immunity, National Clinical Research Center for Child Health and Disorders, Ministry of Education Key Laboratory of Child Development and Disorders, Children’s Hospital of Chongqing Medical University, Chongqing, China

**Keywords:** breastfeeding duration, systemic juvenile idiopathic arthritis, mediation analysis, restricted cubic spline models, juvenile idiopathic arthritis

## Abstract

**Background:**

Systemic juvenile idiopathic arthritis (sJIA) is an autoinflammatory subtype of JIA with distinct immunopathogenic mechanisms. Early-life nutritional exposures such as breastfeeding may influence immune development and inflammatory disease risk, yet evidence in sJIA remains limited. Therefore, this study aimed to examine the association between breastfeeding duration and the likelihood of developing sJIA versus non-sJIA, and to assess whether systemic inflammatory markers mediate this relationship.

**Methods:**

In this cross-sectional study, we included 450 children diagnosed with JIA from 2018 to 2024 at Children’s Hospital of Chongqing Medical University. Breastfeeding duration was retrospectively collected, and patients were classified into sJIA and non-sJIA groups. Multivariable logistic regression and restricted cubic spline models were used to assess the association between breastfeeding duration and the risk of sJIA. Mediation analysis was conducted to quantify the indirect effects of inflammatory mediators on this association.

**Results:**

Among 450 patients with JIA, those with sJIA (*n* = 150) had significantly shorter breastfeeding duration than non-sJIA patients (*n* = 300) (mean 6.3 vs. 9.1 months, *p* < 0.001). sJIA cases also showed higher levels of inflammatory markers, including neutrophil count, C-reactive protein (CRP), erythrocyte sedimentation rate, and white blood cell (WBC). Multivariable logistic regression confirmed that shorter breastfeeding duration was independently associated with higher odds of sJIA (adjusted OR = 0.86; 95% CI: 0.77–0.96; *p* = 0.009). Restricted cubic spline analysis revealed a non-linear inverse relationship between breastfeeding duration and sJIA likelihood, with an inflection point near 7.5 months. Mediation analysis indicated that neutrophil count, WBC count, and CRP partially mediated the relationship between breastfeeding and sJIA, accounting for 26.5, 25.8, and 12.4% of the total effect, respectively.

**Conclusion:**

Shorter breastfeeding duration is associated with a higher probability of sJIA, and this relationship may be partially mediated by systemic inflammatory status. These findings highlight the potential role of early-life nutritional exposures in promoting autoinflammatory disease expression and support further prospective investigations.

## Introduction

Juvenile idiopathic arthritis (JIA) is one of the most common rheumatic diseases in children, with disease onset typically occurring before the age of 16 (1, 2). According to the International League of Associations for Rheumatology (ILAR), JIA is classified into seven mutually exclusive subtypes based on clinical manifestations observed within the first 6 months following disease onset (3). Among these, systemic JIA (sJIA) and non-systemic JIA differ in their underlying immunopathogenic mechanisms. sJIA is currently regarded as a prototypical autoinflammatory disorder, primarily driven by dysregulation of the innate immune system. It is characterized by hyperactivation of the IL-1/IL-6 signaling pathways, increased levels of endogenous Toll-like receptor ligands such as S100 proteins, and a heightened susceptibility to macrophage activation syndrome (MAS) (4, 5). In contrast, non-sJIA subtypes are considered classical autoimmune diseases, marked by Th1/Th17-skewed adaptive immune responses, impaired regulatory T cell (Treg) function, and a breakdown of immunological tolerance (6).

Infancy represents a critical window for immune system development, during which nutritional exposures—most notably breastfeeding—can exert long-lasting immunomodulatory effects (7). Human milk is rich in a variety of bioactive components, including cytokines, human milk oligosaccharides, maternal antibodies, and microbiota-derived metabolites (8). These constituents work synergistically to provide passive immune protection, shape both innate and adaptive immune responses, promote the colonization of gut microbiota, and maintain mucosal barrier integrity (9–11). In view of these benefits, the American Academy of Pediatrics recommends exclusive breastfeeding for the first 6 months of life, followed by continued breastfeeding for at least 1 year (12, 13). Numerous epidemiological studies have shown that breastfeeding is associated with a reduced risk of various immune-mediated diseases, including classical autoimmune conditions such as type 1 diabetes, as well as atopic diseases such as asthma and atopic dermatitis (14–17).

However, data on the impact of breastfeeding in autoinflammatory conditions such as sJIA remain scarce, and no study to date has systematically compared its effects across different JIA subtypes. It is noteworthy that sJIA is often associated with more severe systemic disease and more complex treatment management compared to non-systemic forms. Children with sJIA are at risk of life-threatening complications, including MAS and sJIA-associated lung disease, for which current biologic therapies offer only limited protection (18, 19). Therefore, identifying modifiable early-life factors such as breastfeeding may provide preliminary evidence to raise awareness of potential protective factors in sJIA.

Against this backdrop, the present study investigates whether breastfeeding duration displays a restricted cubic spline relationship with the risk of developing sJIA versus non-sJIA, modeled with restricted cubic splines to capture potential non-linear effects—and assesses whether systemic inflammatory markers, including C-reactive protein (CRP), erythrocyte sedimentation rate (ESR) and peripheral-blood leukocyte counts, mediate these associations. Clarifying these potential relationships may deepen our understanding of how early nutritional exposures influence susceptibility to autoinflammatory diseases and ultimately inform preventive and therapeutic strategies in pediatric rheumatology.

## Methods

### Study design and participants

Patients diagnosed with JIA who were treated at the Children’s Hospital of Chongqing Medical University between January 2018 and December 2024 were collected. Patients were eligible for inclusion if they met the following criteria: (i) patients diagnosed with JIA according to the 2001 classification criteria of the International League of Associations for Rheumatology (ILAR) (3); (ii) disease onset before the age of 16 years; (iii) no prior treatment with glucocorticoids, conventional DMARDs, or biologic agents at the time of initial sample collection; (iv) availability of complete breastfeeding history; (v) sufficient baseline clinical data. Exclusion criteria were: (i) coexisting autoimmune or infectious diseases; (ii) history of malignancy; (iii) incomplete clinical or laboratory records.

According to the ILAR classification, JIA comprises seven subtypes. In this study, we categorized cases into systemic and non-systemic forms based on their distinct clinical and immunological features, as described by Lee et al. (20). Systemic JIA is uniquely characterized by an autoinflammatory pathogenesis predominantly driven by innate immunity, with early systemic manifestations such as quotidian fever, evanescent rash, and serositis. In contrast, the other six subtypes are primarily autoimmune in nature, mediated by adaptive immunity, and present mainly with synovial inflammation and articular symptoms. This dichotomization was adopted to align with our primary research focus on the innate immunity–driven mechanisms of sJIA and to maintain sufficient statistical power. Applying the full seven-category ILAR classification would have resulted in small sample sizes for several subtypes, thereby reducing statistical efficiency and increasing the risk of type II errors.

**Figure 1:**
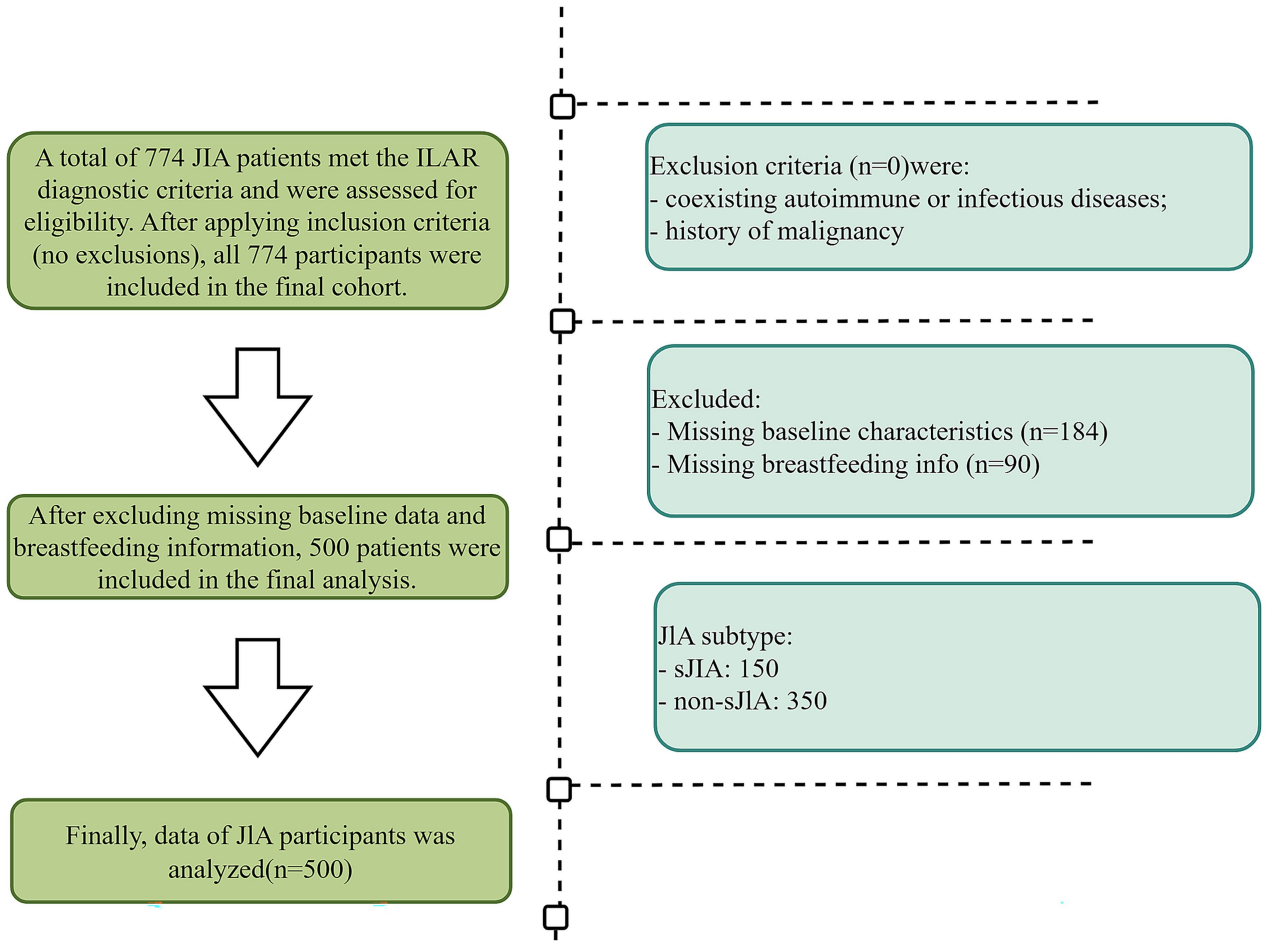
Flow diagram of the study population.

Following application of the study’s inclusion and exclusion criteria, 450 JIA patients were included 150 sJIA and 300 non-sJIA (Figure 1).

### Data collection

Clinical information was obtained retrospectively via the hospital’s big data platform and, partial information was obtained through telephone follow-up. Extracted variables included Demographic and perinatal information: chronological age (years), Breastfeeding time (months) and breastfeeding duration was defined as the total number of months the infant received any amount of breast milk, number of pregnancies (gravidity), number of births (parity), and sex (*n*, %). Hospitalization and disease characteristics: hospitalization (days), symptom onset to visit (days from symptom onset to first hospital visit), time since onset of pain symptoms (days), and raw count of painful joints. Laboratory parameters: white blood cell (WBC), erythrocyte sedimentation rate (ESR), absolute monocyte, platelet, C-reactive protein (CRP), absolute lymphocyte, and absolute neutrophil. Cytokine profiles: interleukin (IL)-4, IL-8, IL-12p70, IL-2, IL-5, tumor necrosis factor-*α* (TNF-α), interferon-γ (IFN-γ), IL-17A, IL-6, IL-1β, and interferon-α (IFN-α).

### Statistical analysis

Statistical analyses were carried out in R 3.5. Continuous variables, reported as mean ± SD or median (IQR), were compared with one-way ANOVA or, when normality assumptions were not met, with the corresponding non-parametric alternative. Categorical variables were compared using Pearson’s chi-square test (21). *p* < 0.05 in the univariate screen were advanced to a multivariable logistic model. Findings are summarized as odds ratios (ORs) with 95% confidence intervals (CIs). Model discrimination was quantified via receiver-operating characteristic (ROC) analysis and its area under the curve (AUC) (22). Breastfeeding duration was modeled as a continuous variable using restricted cubic splines (knots at the 10th, 50th, and 90th percentiles) to assess potential non-linear associations with sJIA risk (23).

Mediation analysis was performed using the “mediation” R package to explore the indirect effects of CRP, neutrophil, and WBC counts on the association between breastfeeding duration and sJIA, adjusting for age, sex, and symptom duration. Sensitivity analyses were conducted using 1,000 bootstrap iterations. And, *p* ≤ 0.05 is considered to be statistically significant.

### Ethical considerations

Ethical clearance was obtained from the Institutional Review Board of the Children’s Hospital of Chongqing Medical University (Approval No. (2023)IRB(STUDY) No. 351). As a retrospective analysis based solely on existing medical records from prior clinical visits, the study posed no additional risk to the participants.

## Results

### Baseline characteristics

The analytical cohort comprised 450 patients diagnosed with JIA, comprising 150 patients with sJIA (sJIA; 86 females, 64 males; mean age: 8.78 ± 3.58 years) and 300 patients with non-sJIA (non-sJIA; 170 females, 130 males; mean age: 9.68 ± 3.52 years). As summarized in Table 1, there were no statistically significant differences between the two groups in terms of age (*p* = 0.161), sex distribution (*p* = 0.893), parity (*p* = 0.406), or gravidity (*p* = 0.149).

**Table 1:** Baseline data of sJIA and non-sJIA patients.

Variables	Total (*n* = 450)	Non-sJIA (*n* = 300)	sJIA (*n* = 150)	*P*
Age (years), Mean ± SD	9.38 ± 3.56	9.68 ± 3.52	8.78 ± 3.58	0.161
Sex, *n* (%)				0.893
Female	256 (56.89)	170 (56.67)	86 (57.33)	
Male	194 (43.11)	130 (43.33)	64 (42.67)	
Production times, Mean ± SD	1.56 ± 0.69	1.54 ± 0.71	1.60 ± 0.65	0.406
Pregnancy frequency, Mean ± SD	1.92 ± 1.13	1.87 ± 1.03	2.04 ± 1.30	0.149
breastfeeding duration (months), Mean ± SD	8.17 ± 4.30	9.10 ± 3.71	6.30 ± 4.78	<0.001
Hospitalization (days), Mean ± SD	10.67 ± 6.85	9.21 ± 6.33	13.59 ± 6.92	<0.001
Symptom onset to visit (days), Mean ± SD	240.98 ± 432.55	319.48 ± 461.35	84.49 ± 315.97	<0.001
Painful joint, Mean ± SD	2.47 ± 1.96	2.73 ± 1.94	1.95 ± 1.91	<0.001
Monocyte (absolute), Mean ± SD	0.39 ± 0.20	0.38 ± 0.15	0.45 ± 0.26	0.061
Platelet, Mean ± SD	369.15 ± 125.31	357.40 ± 112.66	392.64 ± 144.97	0.010
Lymphocyte (absolute), Mean ± SD	2.46 ± 1.08	2.49 ± 0.92	2.41 ± 1.35	0.482
Neutrophil (absolute), Mean ± SD	6.60 ± 5.16	4.82 ± 2.83	10.18 ± 6.69	<0.001
Erythrocyte sedimentation rate, Mean ± SD	45.31 ± 33.07	37.31 ± 29.47	61.76 ± 34.05	<0.001
White blood cell, Mean ± SD	9.61 ± 5.38	7.81 ± 3.23	13.21 ± 6.85	<0.001
C-reactive protein, Mean ± SD	39.78 ± 58.81	22.24 ± 40.52	75.82 ± 72.70	<0.001
Interleukin-4, Mean ± SD	1.20 ± 3.00	1.24 ± 3.36	1.11 ± 2.04	0.725
Interleukin-17A, Mean ± SD	8.54 ± 50.16	10.49 ± 61.69	4.84 ± 8.06	0.422
Interleukin-2, Mean ± SD	1.31 ± 3.00	1.44 ± 3.28	1.09 ± 2.48	0.345
Tumor necrosis factor-alpha, Mean ± SD	3.93 ± 10.91	4.74 ± 12.77	2.81 ± 7.55	0.100
Interleukin-6, Mean ± SD	77.32 ± 292.89	70.75 ± 344.33	89.46 ± 160.01	0.600
Interferon-gamma, Mean ± SD	3.37 ± 15.92	4.36 ± 20.60	2.18 ± 6.77	0.299
Interleukin-8, Mean ± SD	7.14 ± 8.87	5.26 ± 4.92	8.87 ± 11.11	0.007
Interleukin-1 beta, Mean ± SD	2.11 ± 3.49	1.95 ± 3.29	2.28 ± 3.70	0.539
Interleukin-5, Mean ± SD	0.54 ± 0.69	0.49 ± 0.59	0.58 ± 0.78	0.398
Interferon-alpha, Mean ± SD	3.76 ± 6.49	4.15 ± 6.94	1.43 ± 0.85	0.448
Interleukin-12p70, Mean ± SD	2.02 ± 4.81	1.99 ± 6.08	2.04 ± 3.23	0.955

Notably, patients with sJIA had significantly shorter breastfeeding duration (*p* < 0.001), shorter symptom onset to visit (p < 0.001), shorter durations of pain-related symptoms (p < 0.001), and fewer painful joints (*p* < 0.001), but a longer hospitalization days compared to those with non-sJIA. Inflammatory markers were markedly elevated in the sJIA group, including higher neutrophil counts (*p* < 0.001), ESR (*p* < 0.001), WBC count (*p* < 0.01), CRP (*p* < 0.001), and platelet count (*p* = 0.010). Cytokine profiling revealed significantly higher levels of IL-8 (*p* = 0.007), while other cytokines such as IL-4, IL-2, IL-6, IL-17A, TNF-α, and IFN-γ showed no significant differences (all *p* > 0.050).

### Univariate and multivariable logistic regression analysis

Univariate logistic regression identified several significant predictors of sJIA (Table 2). These included shorter breastfeeding duration (OR = 0.82, 95% CI: 0.77–0.87, *p* < 0.001), symptom onset to visit (OR = 0.99, 95% CI: 0.99–0.99, *p* < 0.001), fewer painful joints (OR = 0.79, 95% CI: 0.71–0.89, *p* < 0.001), longer hospitalization days (OR = 1.11, 95% CI: 1.07–1.16, *p* < 0.001), and elevated inflammatory parameters including neutrophil count, ESR, WBC count, CRP, and IL-8.

**Table 2:** Univariate and multivariable logistic regression analysis in this study.

Variables	Univariate logistic regression analysis		Multivariable logistic regression analysis	
	OR (95%CI)	*P*	OR (95%CI)	*P*
Breastfeeding duration	0.82 (0.77 ~ 0.87)	<0.001	0.86 (0.77 ~ 0.96)	0.009
Symptom onset to visit (days)	0.99 (0.99 ~ 0.99)	<0.001	0.99 (0.99 ~ 0.99)	0.003
Hospitalization (days)	1.11 (1.07 ~ 1.16)	<0.001	1.10 (1.01 ~ 1.20)	0.023
Painful joint count	0.79 (0.71 ~ 0.89)	<0.001	0.78 (0.62 ~ 0.97)	0.024
Neutrophil (absolute)	1.33 (1.25 ~ 1.43)	<0.001	1.42 (0.92 ~ 2.18)	0.113
Erythrocyte sedimentation rate	1.02 (1.02 ~ 1.03)	<0.001	1.00 (0.99 ~ 1.02)	0.743
White blood cell	1.29 (1.22 ~ 1.37)	<0.001	0.82 (0.55 ~ 1.21)	0.316
C-reactive protein	1.02 (1.01 ~ 1.02)	<0.001	1.02 (1.01 ~ 1.03)	0.033
Interleukin-8	1.07 (1.01 ~ 1.12)	0.017	1.05 (0.99 ~ 1.12)	0.131

In the multivariate model, breastfeeding duration remained independently associated with reduced odds of sJIA (OR = 0.86, 95% CI: 0.77–0.96, *p* = 0.009). Other independent predictors included shorter symptom duration (OR = 0.99, *p* = 0.003), fewer painful joints (OR = 0.78, *p* = 0.024), longer hospital stay (OR = 1.10, *p* = 0.023), and higher CRP levels (OR = 1.02, *p* = 0.033).

**Figure 2:**
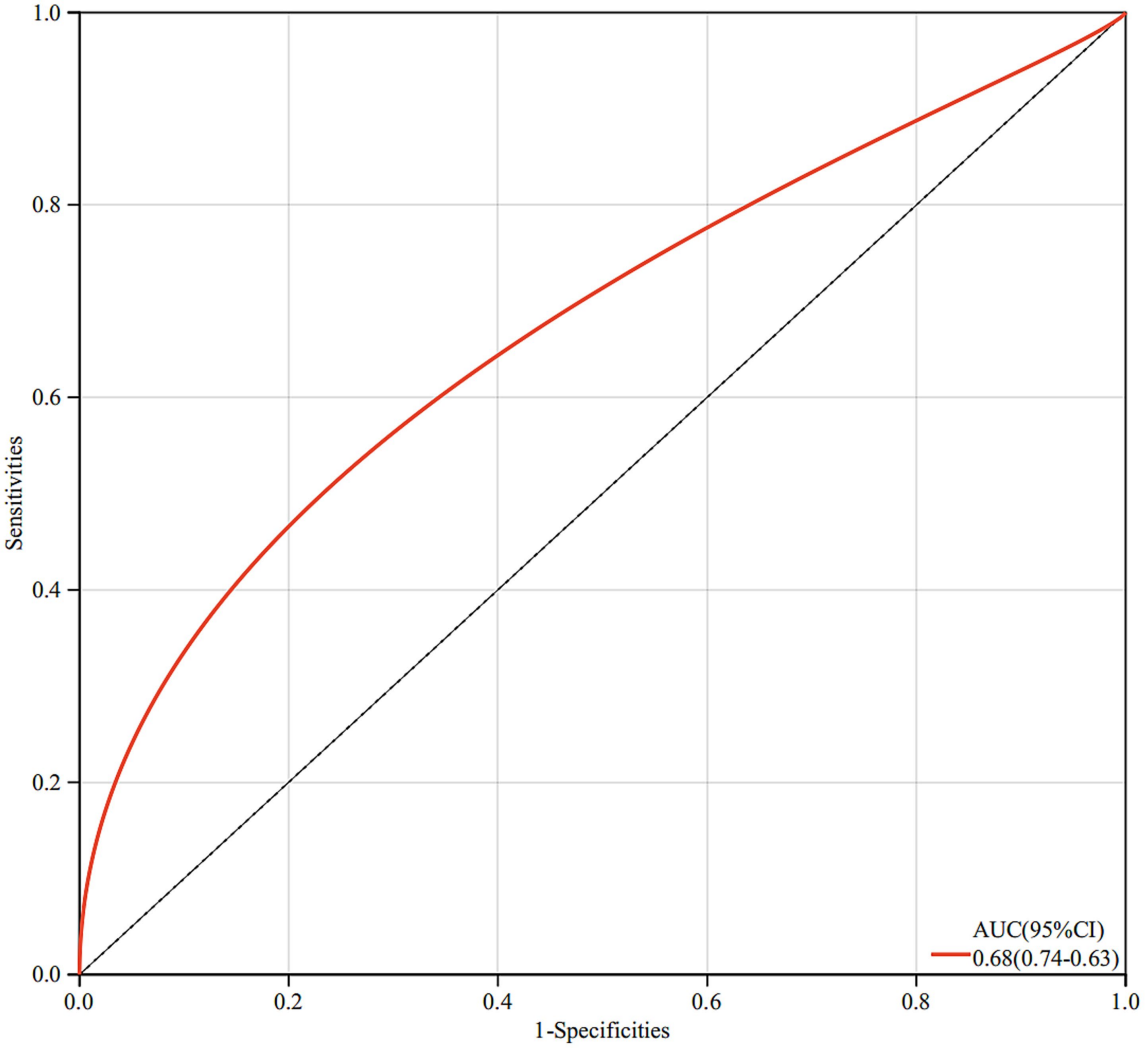
The ROC curve yielding an AUC between breastfeeding duration and sJIA.

The final model demonstrated good discriminative ability, with a ROC curve yielding an AUC of 0.68, suggesting moderate predictive performance of breastfeeding duration in differentiating sJIA from non-sJIA (Figure 2).

### Restricted cubic spline models relationship between breastfeeding duration and sJIA

**Figure 3:**
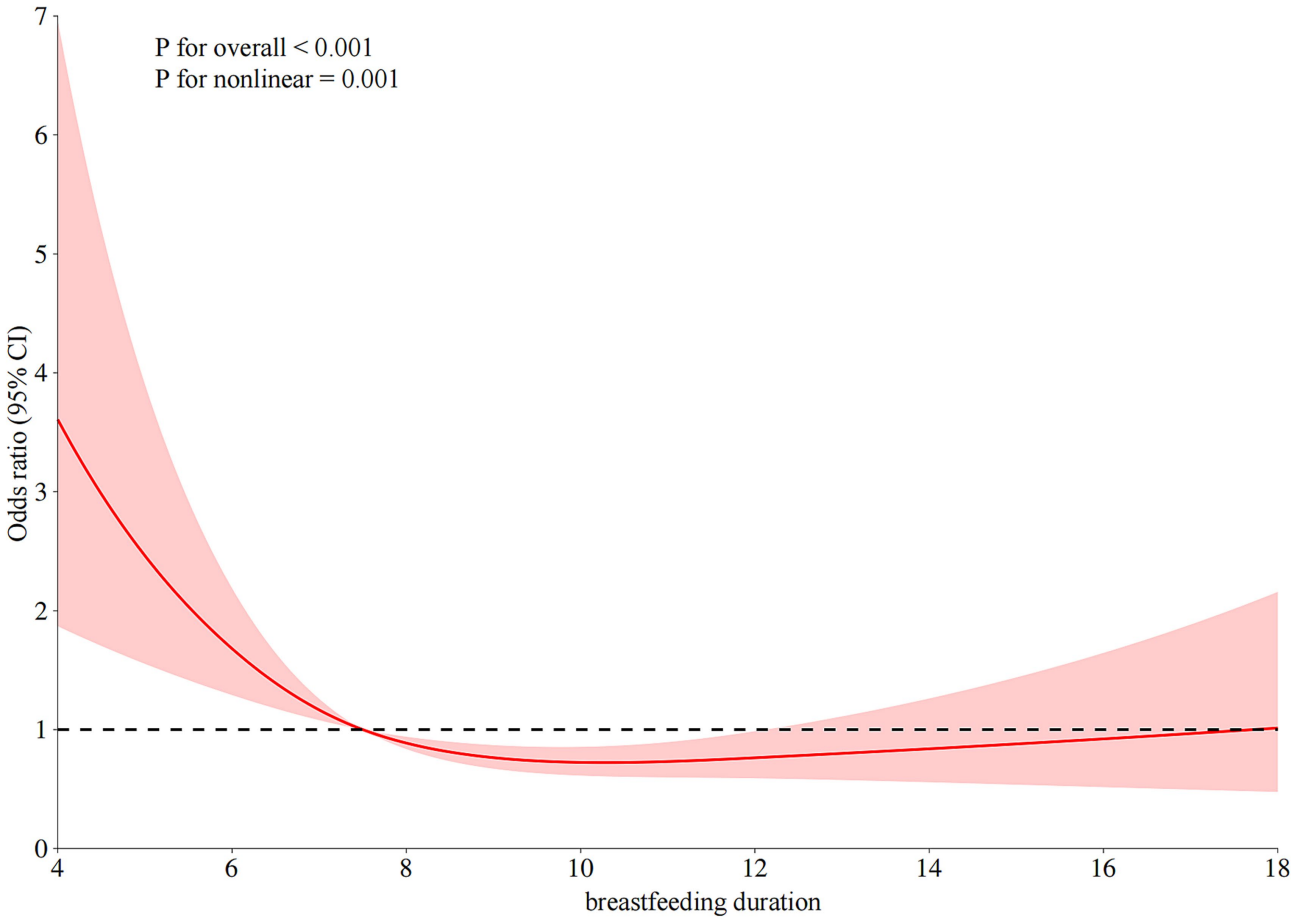
The restricted cubic spline relationships between breastfeeding duration (months) and sJIA.

Restricted cubic spline regression analysis revealed a non-linear inverse association between breastfeeding duration and the risk of developing sJIA (*P* for overall < 0.001; *P* for non-linearity = 0.001). As shown in Figure 3, at approximately 7.5 months, the OR approached 1, indicating a potential inflection point in the association. Before 7.5 months, the OR was greater than 1, suggesting that shorter breastfeeding duration was associated with a higher occurrence of sJIA. Between 7.5 and approximately 13 months, the OR remained below 1, indicating a protective association with extended breastfeeding duration.

### Correlation between breastfeeding duration and various clinical parameters

**Figure 4:**
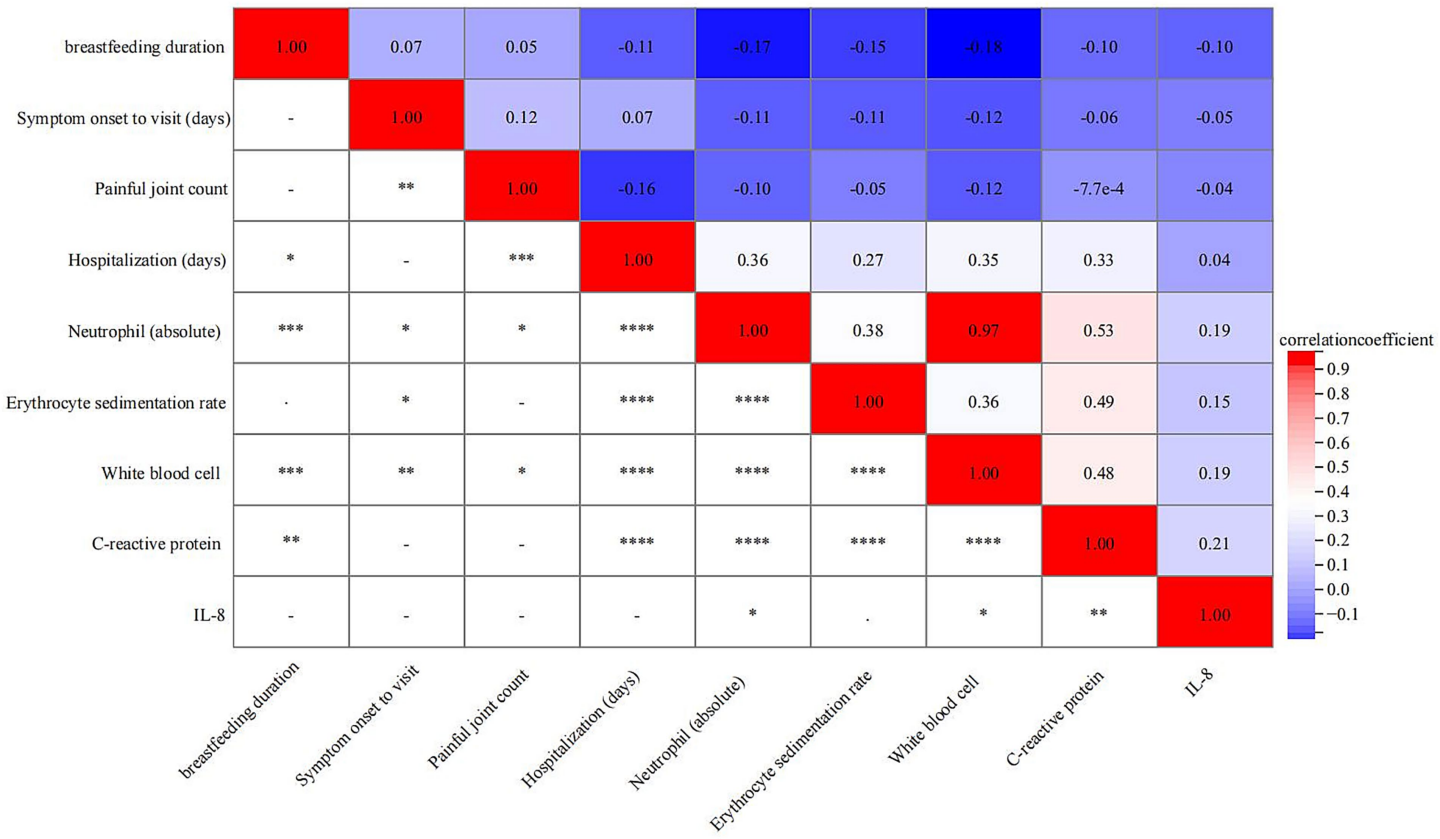
Correlation between breastfeeding duration and various clinical parameters. *< 0.05, ** < 0.01, *** < 0.001, ****<0.0001.

Spearman correlation analysis demonstrated that breastfeeding duration was negatively correlated with several inflammatory markers. Specifically, significant inverse correlations were observed with neutrophil count (*r* = −0.18, *p* < 0.001), WBC count (*r* = −0.17, *p* < 0.001), and CRP also showed a weaker yet statistically significant negative correlation (*r* = −0.10, *p* < 0.010) (Figure 4).

### Mediation analysis between breastfeeding duration and sJIA

Mediation analysis suggested that the protective association between breastfeeding duration and sJIA risk was partially mediated by systemic inflammatory markers. Among these, neutrophil count and WBC count accounted for 26.48 and 25.76% of the total effect, respectively, while CRP mediated 12.43% (Table 3). Both the indirect and direct effects remained statistically significant in all models (*p* < 0.001), indicating that breastfeeding influences sJIA risk through both inflammation-dependent and independent pathways.

**Table 3:** Mediation analysis between breastfeeding duration and sJIA.

Variables	Effect	Estimate	Lower	Upper	β (95%CI)	*P*	Mediation
C-reactive protein							
	Indirect	0	−0.01	0	−0.00 (−0.01 ~ −0.00)	0.04	12.43
	Direct	−0.04	−0.04	−0.03	−0.04 (−0.04 ~ −0.03)	<0.001	87.57
	Total	−0.04	−0.05	−0.03	−0.04 (−0.05 ~ −0.03)	<0.001	100
Neutrophil (absolute)							
	Indirect	−0.01	−0.01	−0.01	−0.01 (−0.01 ~ −0.01)	<0.001	26.48
	Direct	−0.03	−0.04	−0.02	−0.03 (−0.04 ~ −0.02)	<0.001	73.52
	Total	−0.04	−0.04	−0.03	−0.04 (−0.04 ~ −0.03)	<0.001	100
White blood cell							
	Indirect	−0.01	−0.01	−0.01	−0.01 (−0.01 ~ −0.01)	<0.001	25.76
	Direct	−0.03	−0.04	−0.02	−0.03 (−0.04 ~ −0.02)	<0.001	74.24
	Total	−0.04	−0.04	−0.03	−0.04 (−0.04 ~ −0.03)	<0.001	100

## Discussion

Systemic juvenile idiopathic arthritis is a clinically and immunologically distinct subtype within the JIA spectrum, characterized by innate immune hyperactivation and systemic inflammation, and is increasingly regarded as an autoinflammatory disorder (24). Unlike other JIA subtypes, sJIA responds poorly to traditional immunosuppressive therapies and is associated with serious complications such as macrophage activation syndrome. Identifying modifiable early-life factors that influence its development is therefore of urgent clinical relevance (25). In this retrospective cohort study, we found that shorter breastfeeding duration was significantly associated with an increased risk of sJIA, but not non-systemic JIA. This relationship showed a non-linear dose–response pattern and was partially mediated by systemic inflammatory markers, particularly neutrophil and white blood cell counts. To our knowledge, this is the first study to implicate breastfeeding as a potential early-life protective factor specifically for sJIA, suggesting that early nutritional exposures may modulate the risk of autoinflammatory disease via inflammatory pathways.

Breastfeeding has long been recognized for its immunomodulatory properties, primarily attributed to its rich content of bioactive components such as immunoglobulins, cytokines, and microbiota-regulating oligosaccharides. These elements not only enhance mucosal barrier function but also shape systemic immune responses during early infancy, thereby influencing long-term immunological homeostasis (7–11). Our findings align with those of a clinical study conducted in Fortaleza, Brazil, which cross-sectionally evaluated 91 patients with JIA between May 2015 and April 2016. That study found that breastfeeding beyond 6 months was significantly associated with lower disease activity and fewer permanent joint deformities, particularly in socioeconomically disadvantaged populations with high breastfeeding prevalence (26). In addition, the prospective ABIS cohort study from Sweden further supports this association. Kindgren et al. (27) reported that children breastfed for less than 4 months had a significantly increased risk of developing JIA compared to those breastfed for longer than 4 months (adjusted OR = 3.5, 95% CI: 1.4–8.5; *p* = 0.006). Both shorter durations of exclusive and total breastfeeding were independently associated with higher JIA risk (aOR = 1.3 and 1.2, respectively; *p* < 0.01), even after adjusting for multiple confounders (27). Although these studies did not stratify by JIA subtype, they collectively suggest a general protective role of extended breastfeeding in mitigating chronic inflammatory joint diseases during childhood. In our cohort, this protective association was particularly pronounced in the sJIA. Breastfeeding duration was inversely associated with sJIA risk, whereas no such trend was observed in non-sJIA patients. This finding implies that the immunomodulatory effects of breastfeeding may be especially relevant in autoinflammatory diseases characterized by innate immune dysregulation. Mediation analysis further revealed that neutrophil and white blood cell counts partially mediated this association, suggesting that systemic inflammation may serve as a key mechanistic link between early-life nutritional exposures and sJIA pathogenesis. Moreover, restricted cubic spline modeling demonstrated a clear non-linear dose–response relationship between breastfeeding duration and sJIA risk. An inflection point was observed around 7.5 months, beyond which prolonged breastfeeding continued to reduce risk, albeit with diminishing marginal returns. These results collectively support the hypothesis that early-life nutrition may influence susceptibility to sJIA via modulation of systemic inflammatory tone.

Although the exact mechanisms underlying the protective association between breastfeeding and systemic JIA remain unclear, we speculate that this effect may stem from the influence of breastfeeding on early innate immune development during a critical postnatal window (28). Breast milk contains a complex array of immunologically active components—including secretory IgA, anti-inflammatory cytokines such as IL-10 and TGF-β, and human milk oligosaccharides (HMOs)—that work synergistically to support immune homeostasis (29). For instance, secretory IgA contributes to mucosal barrier integrity by preventing microbial translocation across the gut epithelium (30), thereby limiting systemic exposure to pathogen-associated molecular patterns (PAMPs), which can overactivate innate immune sensors such as Toll-like receptors (TLRs) (31). Meanwhile, cytokines like IL-10 and TGF-β actively dampen pro-inflammatory signaling pathways and promote regulatory immune phenotypes, reducing the likelihood of chronic inflammation (32). HMOs play a critical role in shaping the infant gut microbiota toward a more tolerogenic and less pro-inflammatory composition, which in turn affects systemic immune tone (33). These mechanisms are particularly relevant to the pathogenesis of sJIA, which is characterized by neutrophil-driven inflammation (34), elevated CRP, and dysregulation of myeloid cell responses (35). The partial mediation we observed through peripheral neutrophil and total WBC counts suggests that breastfeeding may reduce the baseline priming or expansion of these inflammatory cell populations. This hypothesis aligns with the idea that early-life nutritional exposures help set the inflammatory “set point” of the innate immune system, which could tip the balance toward or away from autoinflammatory disease expression in genetically predisposed children.

From a clinical and public health perspective, our findings underscore the potential of breastfeeding as a modifiable early-life exposure that may reduce the risk of severe autoinflammatory diseases such as sJIA. In particular, populations with limited access to early rheumatologic care may benefit from enhanced breastfeeding support as part of broader disease prevention strategies. Furthermore, the non-linear dose–response relationship we identified offers a valuable benchmark—suggesting that 7.5 months of breastfeeding may confer meaningful immunological protection, without requiring indefinite duration.

Our study also has some limitations. First, although breastfeeding is recognized for its immunomodulatory potential, the reported duration was based on retrospective maternal recall, which may introduce recall and classification bias. Inflammatory markers such as neutrophil and white blood cell counts were measured post-diagnosis, complicating association inference in mediation analyses due to uncertain temporal precedence. Additionally, other early-life exposures—such as perinatal microbial contact and caregiver skin-to-skin interaction—were not assessed. And, as cytokine levels were measured after diagnosis, we cannot fully establish whether the observed mediation effect reflects a causal pathway from breastfeeding duration to disease risk via cytokine modulation, or whether cytokine alterations are secondary to established disease. Thus, the mediation analysis should be interpreted as indicative of association rather than causality. Prospective studies with pre-diagnostic biomarker data are needed to confirm these relationships. Another limitation is that breastfeeding type (exclusive versus partial) was not distinguished in our dataset. As these feeding patterns may differ in their immunological effects, the use of total breastfeeding duration alone may obscure specific associations. Future studies incorporating detailed feeding pattern data are warranted to further clarify these relationships. In addition, other early-life exposures—including timing of complementary food introduction, daycare attendance, and antibiotic use—were not assessed. As these factors may affect immune maturation and potentially confound the observed associations, their omission should be considered when interpreting our results. Future studies should incorporate these variables to allow more comprehensive adjustment for early-life environmental influences. Lastly, the single-center retrospective design and limited sample size may affect generalizability. Future prospective, multi-center studies with detailed nutritional and immunological profiling are needed to validate and expand upon these findings.

In summary, our findings suggest that longer breastfeeding duration may be associated with a lower risk of developing systemic juvenile idiopathic arthritis (sJIA), but not non-systemic forms of the disease. This association appears to follow a non-linear dose–response pattern and may be partially mediated by systemic inflammatory markers such as neutrophil and white blood cell counts. The results raise the possibility that early-life nutritional exposures could influence the risk of autoinflammatory diseases. Further prospective, subtype-specific research is warranted to clarify these relationships and explore their implications for early prevention strategies.

## Data Availability

The raw data supporting the conclusions of this article will be made available by the authors, without undue reservation.

## References

[1] Luo X Luo X Luo Q Tang X. Disease activity and treatment in patients with juvenile idiopathic arthritis before transfer to adult care: the first survey in China. Front Pediatr. (2025) 13:1535223. doi: 10.3389/fped.2025.1535223 40248020 PMC12004128

[2] Luo Q Hao H Xiwen L Qiu X Liu D Liu Y. UBE2D1 as a key biomarker in systemic juvenile idiopathic arthritis: a new perspective on diagnosis and disease activity assessment. Arthritis Res Ther. (2025) 27:140. doi: 10.1186/s13075-025-03606-8, PMID: 40635084 PMC12239252

[3] Petty RE Southwood TR Manners P Baum J Glass DN Goldenberg J. International league of associations for rheumatology classification of juvenile idiopathic arthritis: second revision, Edmonton, 2001. J Rheumatol. (2004) 31:390–2. 14760812

[4] Kumar S. Systemic juvenile idiopathic arthritis: diagnosis and management. Indian J Pediatr. (2016) 83:322–7. doi: 10.1007/s12098-016-2060-z, PMID: 26916892

[5] Bruck N Schnabel A Hedrich CM. Current understanding of the pathophysiology of systemic juvenile idiopathic arthritis (sJIA) and target-directed therapeutic approaches. Clin Immunol. (2015) 159:72–83. doi: 10.1016/j.clim.2015.04.018 25956529

[6] Lin YT Wang CT Gershwin ME Chiang BL. The pathogenesis of oligoarticular/polyarticular vs. systemic juvenile idiopathic arthritis. Autoimmun Rev. (2011) 10:482–9. doi: 10.1016/j.autrev.2011.02.001, PMID: 21320644

[7] Hamdan TA Alkhateeb S Oriquat G Alzoubi A Ahmed KA. Impact of breastfeeding and formula feeding on immune cell populations and blood cell parameters: an observational study. J Int Med Res. (2024) 52:3000605241307217. doi: 10.1177/03000605241307217, PMID: 39731441 PMC11686727

[8] Ruiz L Espinosa-Martos I García-Carral C Manzano S McGuire MK Meehan CL. What's Normal? Immune profiling of human Milk from healthy women living in different geographical and socioeconomic settings. Front Immunol. (2017) 8:696. doi: 10.3389/fimmu.2017.00696, PMID: 28713365 PMC5492702

[9] Dawod B Marshall JS Azad MB. Breastfeeding and the developmental origins of mucosal immunity: how human milk shapes the innate and adaptive mucosal immune systems. Curr Opin Gastroenterol. (2021) 37:547–56. doi: 10.1097/MOG.0000000000000778, PMID: 34634003 PMC11451935

[10] Huang X Fan X Ying J Chen S. Emerging trends and research foci in gastrointestinal microbiome. J Transl Med. (2019) 17:67. doi: 10.1186/s12967-019-1810-x, PMID: 30819194 PMC6396506

[11] Rogier EW Frantz AL Bruno ME Wedlund L Cohen DA Stromberg AJ. Secretory antibodies in breast milk promote long-term intestinal homeostasis by regulating the gut microbiota and host gene expression. Proc Natl Acad Sci USA. (2014) 111:3074–9. doi: 10.1073/pnas.1315792111, PMID: 24569806 PMC3939878

[12] Li WJ Gao YC Hu X Tan YT Deng JJ Pan HF. Association between breastfeeding and the risk of autoimmune diseases: a systematic review and meta-analysis. Autoimmun Rev. (2025) 24:103801. doi: 10.1016/j.autrev.2025.103801, PMID: 40081726

[13] Belderbos ME Houben ML van Bleek GM Schuijff L van Uden NOP Bloemen-Carlier EM. Breastfeeding modulates neonatal innate immune responses: a prospective birth cohort study. Pediatr Allergy Immunol. (2012) 23:65–74. doi: 10.1111/j.1399-3038.2011.01230.x, PMID: 22103307

[14] Mimouni Bloch A Mimouni D Mimouni M Gdalevich M. Does breastfeeding protect against allergic rhinitis during childhood? A meta-analysis of prospective studies. Acta Paediatr. (2002) 91:275–9. doi: 10.1080/08035250252833914, PMID: 12022298

[15] Gerstein HC. Cow's milk exposure and type I diabetes mellitus. A critical overview of the clinical literature. Diabetes Care. (1994) 17:13–9. doi: 10.2337/diacare.17.1.13, PMID: 8112184

[16] Gdalevich M Mimouni D Mimouni M. Breast-feeding and the risk of bronchial asthma in childhood: a systematic review with meta-analysis of prospective studies. J Pediatr. (2001) 139:261–6. doi: 10.1067/mpd.2001.117006, PMID: 11487754

[17] Gdalevich M Mimouni D David M Mimouni M. Breast-feeding and the onset of atopic dermatitis in childhood: a systematic review and meta-analysis of prospective studies. J Am Acad Dermatol. (2001) 45:520–7. doi: 10.1067/mjd.2001.114741, PMID: 11568741

[18] Woerner A von Scheven-Gête A Cimaz R Hofer M. Complications of systemic juvenile idiopathic arthritis: risk factors and management recommendations. Expert Rev Clin Immunol. (2015) 11:575–88. doi: 10.1586/1744666X.2015.1032257, PMID: 25843554

[19] Cimaz R. Systemic-onset juvenile idiopathic arthritis. Autoimmun Rev. (2016) 15:931–4. doi: 10.1016/j.autrev.2016.07.004 27392503

[20] Lee PY Schulert GS Canna SW Huang Y Sundel J Li Y. Adenosine deaminase 2 as a biomarker of macrophage activation syndrome in systemic juvenile idiopathic arthritis. Ann Rheum Dis. (2020) 79:225–31. doi: 10.1136/annrheumdis-2019-216030, PMID: 31707357 PMC7385992

[21] Luo Q Qin L Zhang Y Yang X Wang H. Relationship between serum uric acid and hypertension in patients with primary Sjögren's syndrome: a retrospective cohort study. J Clin Hypertens (Greenwich). (2022) 24:1026–34. doi: 10.1111/jch.14541, PMID: 35809227 PMC9380167

[22] Obuchowski NA Bullen JA. Receiver operating characteristic (ROC) curves: review of methods with applications in diagnostic medicine. Phys Med Biol. (2018) 63: 07TR01. doi: 10.1088/1361-6560/aab4b1, PMID: 29512515

[23] Lusa L Ahlin Č. Restricted cubic splines for modelling periodic data. PLoS One. (2020) 15:e0241364. doi: 10.1371/journal.pone.0241364, PMID: 33112926 PMC7592770

[24] Prakken B Albani S Martini A. Juvenile idiopathic arthritis. Lancet. (2011) 377:2138–49. doi: 10.1016/S0140-6736(11)60244-4 21684384

[25] Hinze CH Foell D Kessel C. Treatment of systemic juvenile idiopathic arthritis. Nat Rev Rheumatol. (2023) 19:778–89. doi: 10.1038/s41584-023-01042-z 37923864

[26] Rocha FAC Landim JIVD Nour ML Filho VFP da Rocha LN da Silva MFC. Long-term breastfeeding influences disease activity in a low-income juvenile idiopathic arthritis cohort. Clin Rheumatol. (2019) 38:2227–31. doi: 10.1007/s10067-019-04582-6 31062254

[27] Kindgren E Fredrikson M Ludvigsson J. Early feeding and risk of juvenile idiopathic arthritis: a case control study in a prospective birth cohort. Pediatr Rheumatol Online J. (2017) 15:46. doi: 10.1186/s12969-017-0175-z, PMID: 28549465 PMC5446703

[28] König RS Albrich WC Kahlert CR Bahr LS Löber U Vernazza P. The gut microbiome in Myalgic encephalomyelitis (ME)/chronic fatigue syndrome (CFS). Front Immunol. (2022) 12:628741. doi: 10.3389/fimmu.2021.628741 35046929 PMC8761622

[29] Moszak M Szulińska M Bogdański P. You are what you eat-the relationship between diet, microbiota, and metabolic disorders-a review. Nutrients. (2020) 12:1096. doi: 10.3390/nu12041096, PMID: 32326604 PMC7230850

[30] Ni Z Wang S Li Y Zhou L Zhai D Xia D. Mapping trends and hotspot regarding gut microbiota and host immune response: a bibliometric analysis of global research (2011-2021). Front Microbiol. (2022) 13:932197. doi: 10.3389/fmicb.2022.932197, PMID: 35958122 PMC9361022

[31] Roberts LE Williams CEC Oni L Barratt J Selvaskandan H. IgA nephropathy: emerging mechanisms of disease. Indian J Nephrol. (2024) 34:297–309. doi: 10.25259/ijn_425_23, PMID: 39156850 PMC11326799

[32] Zhou D Zhong W Fu B Li E Hao L Li Q. Dietary supplementation of mulberry leaf oligosaccharides improves the growth, glucose and lipid metabolism, immunity, and virus resistance in largemouth bass (*Micropterus salmoides*). Front Immunol. (2025) 16:1525992. doi: 10.3389/fimmu.2025.1525992, PMID: 39935475 PMC11811104

[33] Zheng J Xu H Fang J Zhang X. Enzymatic and chemoenzymatic synthesis of human milk oligosaccharides and derivatives. Carbohydr Polym. (2022) 291:119564. doi: 10.1016/j.carbpol.2022.119564, PMID: 35698389

[34] Malengier-Devlies B Bernaerts E Ahmadzadeh K Filtjens J Vandenhaute J Boeckx B. Role for granulocyte Colony-stimulating factor in neutrophilic extramedullary Myelopoiesis in a murine model of systemic juvenile idiopathic arthritis. Arthritis Rheumatol. (2022) 74:1257–70. doi: 10.1002/art.42104, PMID: 35243819

[35] Macaubas C Wong E Zhang Y Nguyen KD Lee J Milojevic D. Altered signaling in systemic juvenile idiopathic arthritis monocytes. Clin Immunol. (2016) 163:66–74. doi: 10.1016/j.clim.2015.12.011, PMID: 26747737 PMC4753112

